# Cognitive after-effects and associated correlates among post-illness COVID-19 survivors: a cross-sectional study, Egypt

**DOI:** 10.1186/s41983-022-00505-6

**Published:** 2022-06-20

**Authors:** Mohamed Abdelghani, Samar A. Atwa, Amira Said, Niveen E. Zayed, Ahmed A. Abdelmoaty, Mervat S. Hassan

**Affiliations:** 1 Department of Psychiatry, College of Medicine, Zagazig University, Zagazig, Sharkia Governorate Egypt; 2 2015–2016 Hubert H. Humphrey Fellowship, Department of Psychology, Virginia Commonwealth University, Richmond, VA USA; 3 Department of Anesthesia and Surgical Intensive Care, College of Medicine, Zagazig University, Zagazig, Egypt; 4 Department of Chest Diseases, College of Medicine, Zagazig University, Zagazig, Egypt; 5 Department of Tropical Medicine, College of Medicine, Zagazig University, Zagazig, Egypt

**Keywords:** COVID-19 survivors, Cognitive impairment, Egypt

## Abstract

**Background:**

COVID-19’s after-effects among survivors are of increased concern. The cognitive aftermath of COVID-19 virus infection was underrated. This study aimed to identify and compare the cognitive impairment (CI) and its correlates among COVID-19 survivors and control subjects. A total of 85 adults who survived COVID-19 virus infection and an equal number of control subjects (matched for age, sex, education, and socioeconomic level) were included in this study. They were recruited from Zagazig University Hospitals, Sharkia Province, Egypt. All subjects were interviewed utilizing a semistructured demographic and clinical checklist, the Montreal Cognitive Assessment (MoCA) test, and the Hospital Anxiety Depression Scale (HADS).

**Results:**

More than half of COVID-19 survivors experienced CI (compared to only 8% of control subjects). Individuals who survived COVID-19 virus infection were more likely to have impairments in visuo-executive functions (OR: 0.3, 95% CI 0.2–0.5), attention (OR: 0.4, 95% CI 0.3–0.7), language (OR: 0.2, 95% CI 0.1–0.5), delayed recall (OR: 0.5, 95% CI 0.4–0.6), and total MoCA Scores (OR: 0.1, 95% CI 0.04–0.2). Among COVID-19 survivors, those who experienced CI were likely to be older (OR: 1.1, 95% CI 1.03–1.2), and of low-to-moderate education (OR: 4.9, 95% CI 1.6–15.1).

**Conclusions:**

CI was prevalent among COVID-19 survivors. The visuo-executive functions, attention, language, and delayed recall were the most affected domains. Older age and lower educational level predicted CI in COVID-19 survivors.

## Background

The novel coronavirus, severe acute respiratory syndrome-related coronavirus-2 (SARS-CoV-2), was initially discovered in November 2019 in Wuhan, China, then the associated COVID-19 disease, rapidly became a global pandemic [1]. With the worldwide spread of the COVID-19 pandemic, various studies had documented the negative impact of COVID-19 virus infection on the mental health and quality of life in a wide array of populations including COVID-19 patients [2], healthcare providers [3], patients with pre-existing chronic diseases [4], and even the general population [5].

With the growing numbers of infected people, there was a simultaneous increase in the number of recovered patients with chronic needs [6]. Moreover, experts had stated that despite their remission, some people continued experiencing symptoms of the illness several weeks or even months after being infected. These individuals, which were referred to as “long-haulers,” had cleared their SARS-CoV-2 infections, yet they were not all symptom-free [7]. Many reports referred to continued fatigue, joint and bone pain, palpitations, headaches, dizziness, and insomnia for many months later [6, 8].

Cognitive after-effects were well reported among survivors during the previous epidemics, including, Influenza A (H1N1), severe acute respiratory syndrome (SARS), and Middle East respiratory syndrome (MERS). Previous studies revealed that most SARS patients had complained of problems of concentration and memory, and sleeping disturbances, indicating cognitive impairment (CI) after SARS infection, as well as emotional instability including increased levels of anxiety and depression that persisted even after recovery from active infection [9, 10]. Although the research about the COVID-19 after-effects was scarce, however, there was increasing evidence that coronaviruses might spread to extra-respiratory organs, notably the central nervous system (CNS) [11–13]. One-third of COVID-19 patients reported neurological symptoms, and delirium was detected in more than 20% of hospitalized patients [14, 15].

The underlying mechanisms involved in CI among COVID-19 survivors were not fully understood; however, it might be multifactorial [16, 17]. These factors included direct viral infection of the nervous system, the systemic inflammatory response to the virus, cerebrovascular ischemia due to endothelial dysfunction or severe coagulopathy, hypoxia caused by the acute respiratory distress syndrome (ARDS) presented in the active stage of infection, peripheral organ dysfunction and, the use of invasive ventilation and sedation along with side effects of drugs used to treat COVID-19 virus infection [18–20]. Regarding the evidence of direct brain infection related to COVID-19 virus infection, sparse neuropathological data of COVID-19 cases reported hypoxic changes and demyelinating lesions [21, 22]. It was postulated that SARS-CoV-2, as the other coronaviruses, would show certain neurotropism, which means the viral ability to invade and live in neural tissue, or bind to the angiotensin-converting enzyme 2 (ACE2) receptor on the neuronal cells, particularly the hippocampus, thus increasing the probability of post-remission cognitive impairment [22, 23]. Besides, animal models showed that brain invasion by SARS-CoV-1 via the olfactory bulb was associated with loss of neuronal cells [24]. Even in the absence of direct brain insult, a “cytokine storm” would be precipitated following severe infection which might accelerate subsequent brain tissue damage [25, 26]. This condition created a state of systemic inflammation, resulting in disruption of the blood–brain barrier (BBB), neural and glial cell damage that would be involved in long-term neuro-cognitive sequelae among survivors [27, 28].

Although the impact on cognitive functions among the recovered cases was well studied in the previous pandemics, little is currently known about the post-illness remission effects of coronavirus infection on the brain and its consequences in terms of cognitive functioning. To our knowledge, this work would be one of the earliest studies, if any, in Egypt and the Middle East countries that assessed the cognitive functions among the COVID-19 survivors. Thus, the present study aimed to investigate the prevalence and risk factors of the cognitive after-effects among individuals who survived the COVID-19 outbreak and their control counterparts in Egypt.

## Methods

A comparative cross-sectional design was used for this study, which was conducted during the period from September 1st to November 29th, 2020, in the outpatient clinics of Zagazig University Hospitals (ZUHs). Applying the Epi Info 6.0, at 80% power of the study, and 95% confidence level, the sample size was calculated to be 170 participants [29]. It included a total of 85 adults who survived the COVID-19 virus infection and an equal number of healthy control subjects who were matched for their age, sex, and educational level had no history of COVID-19 infections and volunteered to participate.

The COVID-19 survivors were consecutively recruited in accordance with the WHO discharge criteria adopted by the committee for the COVID-19 isolation units of ZUHs, Sharkia Province, Egypt. They had a history of COVID-19 virus infection confirmed by Polymerase chain reaction “PCR” test detecting the presence of viral nucleic acid in the nasopharyngeal swab and recovered for at least 1 month or more after either home isolation for individuals presented by mild symptoms, or hospital treatment for those presented with moderate-to-severe symptoms with the assurance of stabilization of their general medical condition. Control subjects were chosen from the first-degree relatives of COVID-19 survivors to exclude the effects of the shared genetic and environmental factors on their mental and physical health status. Subjects, who were younger than 18 or older than 60 years, having major medical or primary psychiatric disorders, illiterate, and who refused to participate, were excluded. All investigators interviewing the subjects in this study were never involved at any stage of the management of COVID-19 survivors during their acute illness.

With insurance of the social distancing and appliance of the appropriate precautions, a face-to-face interview was conducted using the following assessment tools:

### Semi-structured sociodemographic and clinical checklist

It included questions designed to collect sociodemographic data about age, gender, educational level, marital status, residence and occupation, and clinical data about the history of psychiatric and/or physical illnesses.

### Structured Clinical Interview for DSM-5 Axis I Disorders (SCID-5)

The Structured Clinical Interview for DSM-5 Axis I Disorders (SCID-5) was a semi-structured tool that helped diagnose or exclude the presence of co-occurring major acute or chronic primary mental illnesses (e.g., psychosis, dementia, or mental retardation) that would affect the results if present [30]. The validity and reliability of SCID-5 were formerly proved in several studies [31, 32]. All participants were first interviewed with SCID-5, and the subjects, whose history of major psychiatric disorders was irrelevant, continued further psychometric assessment.

### The Montreal Cognitive Assessment (MoCA) test

This test was developed as a brief and sensitive screening tool for assessing cognitive functions, namely, executive and visuospatial functions, naming, memory, attention, language, abstraction, recall, and orientation. The final version of the MOCA was a one-page 30-point test and was scored with 26 as a cutoff, below which, CI was considered (with a recommendation to add 1 point to the total score for elders with less than 12 years of education) [33]. Its Arabic version had 92.3% sensitivity and 85.7% specificity and needed 10 min for administration, and 1 min for scoring [34].

### The Hospital Anxiety Depression Scale (HADS)

This self-report rating scale was used for the assessment of symptoms of depression and anxiety, which were considered as potential covariates in this study [35]. It encompassed 14 items with 7 items for each subscale. In addition, each item was scored on a 4-point Likert scale (ranged from 0 to 3), and the total score for each subscale was the sum of the corresponding seven items (ranged from 0 to 21). The valid cases were considered when their total scores were 11 and higher in each subscale. The Arabic version of HADS, applied in this study, was previously examined for its reliability and validity [36].

The collected data were reviewed, coded, and analyzed utilizing the Statistical Package for Social Science program (SPSS version 18.0). The chi-square test was used to make comparisons between the categorical variables, while the continuous variables were manipulated using the independent sample *t*-test to make comparisons between the means of two groups, and the Mann–Whitney-*U* (MWU) test for data not normally distributed. To obtain odds ratios and 95% confidence intervals of CI among COVID-19 survivors and their control counterparts, the conditional logistic regression analysis was applied. All significant results were considered when their probability was less than 5% (*p* < 0.05).

## Results

A total of 98 individuals who survived COVID-19 virus infection were interviewed, of whom 13 subjects (13%) either refused to participate or did not complete their questionnaires. Thus, those aforementioned subjects and their control family counterparts were excluded from the analysis. The mean age of COVID-19 survivors was 35.95 ± 9.4 years. Most of them were females (81%), married (88%), of low-to-moderate education (62%), and skilled workers (90%). There were no statistically significant differences between COVID-19 survivors and their control counterparts in terms of sociodemographic variables. However, COVID-19 survivors were more likely to experience higher levels of anxiety and depressive symptoms (8.24 ± 4.21, and 8.15 ± 4.17, respectively), compared with their control counterparts (6.62 ± 3.85, and 6.13 ± 3.37, respectively) (*P* value = 0.001, and 0.003, respectively), as illustrated in Table 1.

**Table 1:** Sociodemographic and clinical characteristics of COVID-19 survivors and control subjects

Variable	Cases (n = 85)		Control (n=85)		*t*	*P*-value
	Mean ± SD		Mean ± SD			
Age	35.95 ± 9.40		33.68 ± 9.37		1.58	0.117
	**No**	**%**	**No**	**%**	***χ***^**2**^	
Gender (female)	69	81.2	62	72.9	1.63	0.202
Marital status (married)	75	88.2	66	77.6	3.37	0.066
Residence (rural)	55	64.7	46	54.1	1.976	0.160
Level of education						
Low-to-moderate	53	62.4	48	56.5	0.61	0.435
High	32	37.6	37	43.5		
Occupation						
Skilled	77	90.6	70	82.4	2.65	0.266
Employee	7	8.2	12	14.1		
Unemployed	1	1.2	3	2.4		
	**Mean ± SD**		**Mean ± SD**		**MWU**	
HADS scoring						
Depression	8.24 ± 4.21		6.62 ± 3.85		− 2.95	**0.003**
Anxiety	8.15 ± 4.17		6.13 ± 3.37		− 3.33	**0.001**
					***t***	
Visuo/executive	3.98 ± 0.65		4.51 ± 0.61		− 5.46	**< 0.001**
Naming	2.93 ± 0.26		3.00 ± 0.01		− 2.53	**0.012**
Attention	5.29 ± 0.91		5.74 ± 0.52		− 9.94	**< 0.001**
Language	2.42 ± 0.61		2.80 ± 0.43		− 4.67	**< 0.001**
Abstraction	1.87 ± 0.37		2.00 ± 0.01		− 3.21	**0.002**
Delayed recall	2.84 ± 1.50		4.18 ± 1.06		− 6.72	**< 0.001**
Orientation	6.00 ± 0.01		5.99 ± 0.11		1.00	0.319
	**No**	**%**	**No**	**%**	***χ***^**2**^	
Total MoCA score						
Normal	41	48.2	78	91.8	38.35	**< 0.001**
Impaired	44	51.8	7	8.2		

Bold text indicates statistical significance, where *P*-value < 0.05

Regarding the cognitive functions, the COVID-19 survivors were more likely to have cognitive impairment (CI) than the control subjects (51.8% vs. 7%, *P* value < 0.001). In addition, COVID-19 survivors had a significant decline in all cognitive domains except orientation. The affected cognitive domains were visuo-executive skills, naming, attention, language, abstraction, and delayed recall, as illustrated in Table 1. Even after being adjusted for associated anxiety and depressive symptoms, the COVID-19 survivors, compared to the control subjects, had greater odds of CI, namely, the domains of visuo-executive skills (*P* value < 0.001, OR: 0.3, 95% CI 0.2–0.5), attention (*P* value = 0.002, OR: 0.4, 95% CI 0.3–0.7), language (*P* value < 0.001, OR: 0.2, 95% CI 0.1–0.5), delayed recall (*P* value < 0.001, OR: 0.5, 95% CI 0.4–0.6), and total MOCA scores (*P* value < 0.001, OR: 0.1, 95% CI 0.04–0.2), as illustrated in Table 2.

**Table 2:** Adjusted conditional logistic regression of COVID-19 survivors and control subjects by cognitive functions

Variable	*B*	S.E	Wald	*P*-value	OR	CI (95%)
Visuo/executive	− 1.44	0.06	0.02	**< 0.001**	**0.29**	**0.17**–**0.50**
Naming	− 0.71	0.48	2.22	0.136	0.49	0.19–1.25
Attention	− 0.83	0.27	9.38	**0.002**	**0.44**	**0.26**–**0.74**
Language	− 1.44	0.35	17.14	**< 0.001**	**0.24**	**0.12**–**0.47**
Abstraction	− 1.12	0.66	2.91	0.088	0.33	0.09–1.18
Delayed recall	− 0.74	0.15	25.24	**< 0.001**	**0.48**	**0.36**–**0.64**
Total MoCA score < 26	− 2.31	0.46	25.39	**< 0.001**	**0.10**	**0.04**–**0.24**

Conditional Logistic regression was adjusted for associated depressive and anxiety symptoms

Bold text indicates statistical significance, where 95% confidence intervals do not include the null value (1.00)

Table 3 illustrates the factors associated with CI among COVID-19 survivors. There were significant associations between CI and older age (*t* = − 3.99; *P* value < 0.001), lower-to-moderate education (*χ*^2^ = 14.72; *P* value < 0.001), and associated depressive symptoms (MWU = − 2.00, *P*-value = 0.045). Following logistic regression analysis, it was, however, found that only old age (*P*-value = 0.003, OR: 1.1, 95% CI 1.03–1.2) and low-to-moderate education (*P*-value = 0.005, OR: 4.9, 95% CI 1.6–15.1) were associated with CI in COVID-19 survivors, as illustrated in Table 4.

**Table 3:** Factors associated with cognitive impairment among COVID-19 survivors

Variable	Total MoCA score				*t*	*P*-value
	≥ 26 (n = 41)		< 26 (n = 44)			
	Mean ± SD		Mean ± SD			
Age	32.07 ± 8.11		39.57 ± 9.15		− 3.99	**< 0.001**
	**No**	**%**	**No**	**%**	***χ***^**2**^	
Gender						
Female	32	78.0	37	84.1	0.51	0.476
Male	9	22.0	7	15.9		
Marital status						
Married	34	82.9	41	93.2	2.15	0.143
Not married	7	17.1	3	6.8		
Residence						
Rural	23	56.1	32	72.7	2.57	0.109
Urban	18	48.2	12	27.3		
Level of education						
Low-to-moderate	17	41.5	36	81.8	14.72	**< 0.001**
High	24	58.5	8	18.2		
Occupation						
Skilled	39	95.1	38	86.4	2.20	0.334
Employee	2	4.9	5	11.4		
Unemployed	0	0.0	1	2.3		
Hx of psychiatric illnesses						
Yes	2	4.9	5	11.4	1.18	0.277
No	39	95.1	39	88.6		
Hx of medical illnesses						
Yes	8	19.5	11	25.0	0.37	0.544
No	33	80.5	33	75.0		
COVID-19 symptom severity						
Mild	21	51.2	19	43.2	0.55	0.458
Moderate-to-severe	20	48.8	25	56.8		
Medications received during COVID-19 infection						
Antibiotics	36	97.3	38	92.7	0.85	0.356
Anticoagulants	22	59.5	27	65.9	0.34	0.560
Antimalarial	17	45.9	20	48.8	0.06	0.802
Iverizine	28	75.7	25	61.0	1.93	0.165
Steroids	21	56.8	24	58.5	0.03	0.874
	**Mean ± SD**		**Mean ± SD**		**MWU**	
Home isolation (days)	9.80 ± 9.08		9.70 ± 10.20		− 0.38	0.418
Hospital isolation (days)	5.76 ± 8.71		7.20 ± 9.38		− 0.81	0.707
HADS scoring						
Depression	7.22 ± 4.56		9.02 ± 3.61		− 2.00	**0.045**
Anxiety	7.34 ± 4.65		9.07 ± 3.61		− 1.80	0.072

Bold text indicates statistical significance, where *P* value < 0.05

**Table 4:** Adjusted logistic regression of factors associated with CI in COVID-19 survivors

Variable	*B*	S.E	Wald	*P* value	OR	CI (95%)
Age	0.90	0.03	9.04	**0.003**	**1.09**	**1.03**–**1.16**
Education (lower-to-moderate)	1.60	0.5	7.85	**0.005**	**4.94**	**1.62**–**15.08**
HADS (depression)	0.04	0.07	0.35	0.556	1.04	0.91–1.18

Bold text indicates statistical significance, where 95% confidence intervals do not include null value (1.00)

## Discussion

Most of the individuals suffering from COVID-19 infection were recovered. Recovery was principally defined in terms of remission of respiratory tract symptoms; however, it was not the end of the story for some of those patients. This study assessed the post-remission cognitive effects of COVID-19 virus infection among survivors.

The most striking finding revealed in this study was that the COVID-19 survivors were more likely to have CI than the control subjects. Our results mirrored the previous similar studies. Follow-up of a sample of COVID-19 hospitalized patients, who were diagnosed with delirium during their hospitalization, found that around 40% experienced cognitive deficits 1 month after their discharge [37]. Similarly, a French study stated that approximately one-third of ICU-admitted COVID-19 patients had CI upon their discharge [15]. Interestingly, data from 431,051 participants of the UK Biobank prospective study showed that the only significant factor associated with the risk of the infection was the impaired cognitive functions [38], the issue that would raise the concern about the nature and direction of the relationship between the CI and the COVID19 virus infection, which tended to be bidirectional during the pandemic.

The current study also stated that the most cognitive domains affected among COVID-19 survivors were visuo-executive skills, attention, language, and delayed recall. In line with these findings, two recent studies reported that the survivors of severe COVID-19 infection had lower performances mainly in attention, memory, visuospatial, and executive functions, with relatively preserved orientation and language functions [16, 39]. Furthermore, it was claimed that the occurrence of delirium, as a frequent ICU complication, was associated with poorer cognitive performance, regardless of the length of mechanical ventilation or length of ICU admission [40]. On the other hand, another study concluded that no significant differences were found between COVID-19 survivors and the control group in terms of attention, memory, processing speed, executive functions, and perceptual abilities [41]. This apparent difference would be attributed to the smaller sample size in the former study, as well as, utilizing different assessment tools with different inclusion criteria.

There were several factors associated with CI among COVID-19 survivors. The current study revealed that old age and low-to-moderate education were found to increase the odds of CI among survivors. These findings were consistent with the previous studies conducted during COVID-19 and non-COVID-19 similar pandemics. It was documented that highly educated subjects were more likely to have an increased cognitive reserve that helped them have better-coping strategies against the emergencies, such as the current pandemic [42, 43]. However, an online survey, in the UK, including 84,285 COVID-19 survivors, failed to find an association between age, educational level, and CI [44]. This difference would be attributed to using different methodologies and assessment tools. On the other hand, older age survivors might show long-lasting neuropsychiatric and CI sequelae several months following their remission [45, 46]. Being old would be a significant risk factor for CI among COVID-19 survivors. This might be attributed to many explanations. First, elderly individuals might often have mild cognitive impairment (MCI) [47]. Second, delirium was one of the commonest risk factors of developing subsequent cognitive deficits post-infection among the elderly who were already at increased risk of delirium as a result of underlying neurocognitive deficits [40, 48]. Third, the occurred inflammation would also increase susceptibility to silent infarcts, blood–brain barrier permeability, thrombosis, and coagulopathy, all of which might further propagate neurological injury [49]. Finally, the management-related factors, including patient isolation, long-term ventilation/sedation, comorbid medical conditions, most commonly hypertension, diabetes mellitus, and obesity, would be linked to poorer cognitive performance [50, 51].

The findings of the present study could not be viewed apart from a few limitations. First, there would be a concern that cross-sectional design could not establish a causal relationship between CI and related risk factors. Second, the participants were recruited from a single facility in Sharkia province, which was one of the largest provinces in Egypt, with a relatively small-sized sample, so the generalizability of results should be taken cautiously. Third, the cognitive functions, the main outcome, were assessed by a self-rating scale (MoCA) which would make the results prone to recall bias. Thus, further objective neuropsychological and radiological measures would yield more reliable results. However, to our knowledge, this study, despite these limitations, would be one of the earliest studies in Egypt, if any, to investigate one of the potential aftermaths (i.e., cognitive impairment) among post-remission COVID-19 survivors.

## Conclusions

The current study suggested that CI was prevalent among COVID-19 survivors. Older age and lower educational level were the potential predictors of CI among them. It would be essential to provide convenient neuropsychological rehabilitation to those who might be at high risk, e.g., old age and low educated subjects. Although the research in this area is still in its early stage, this study would provide converging evidence to support the hypothesis that COVID-19 infection was more likely to have negative consequences on cognitive functions that might persist during and following the recovery phase. Further research should investigate the associated biological and neuroimaging changes which might explain the potential CI and other neuropsychological symptoms among COVID-19 survivors.

## Data Availability

The data sets utilized in this study are available upon reasonable request to the corresponding author.

## Abbreviations

CI: Cognitive impairment
HADS: Hospital Anxiety and Depression Scale
MoCA: Montreal Cognitive Assessment
SCID-5: Structured Clinical Interview for DSM-5 Axis I Disorders
